# Basic neonatal resuscitation: retention of knowledge and skills of primary health care workers in Port Harcourt, Rivers State, Southern Nigeria

**DOI:** 10.11604/pamj.2021.38.75.25812

**Published:** 2021-01-22

**Authors:** Datonye Christopher Briggs, Augusta Unoma Eneh, Edward Achinike Daniel Alikor

**Affiliations:** 1 Department of Paediatrics and Child Health, Rivers State University Teaching Hospital, Rivers State, Nigeria,; 2 Department of Paediatrics and Child Health, University of Port Harcourt, Rivers State, Nigeria

**Keywords:** Neonatal resuscitation, skills, knowledge, retention, primary health centres, health care worker

## Abstract

**Introduction:**

birth attendants' retention of knowledge and skills of neonatal resuscitation post-training can prevent birth asphyxia by repeatedly applying neonatal resuscitation guidelines. This study assessed primary healthcare workers' retention of knowledge and skills of basic neonatal resuscitation.

**Methods:**

in 28 primary health centres, 106 birth attendants had their knowledge and skills assessed following a one-day neonatal resuscitation training. The evaluation was before, immediately after training, at three months (a subset of participants) and six months. Paired t-tests were used to compare mean scores at two different evaluation times.

**Results:**

the mean baseline knowledge and skills scores were 35.22% ± 12.90% and 21.40% ± 16.91% respectively. Immediately after training, it increased to 81.48% ± 7.05% and 87.40% ± 13.97% respectively (p=0.0001). At three months, it decreased to 55.37% ± 20.50% and 59.11% ± 25.55% respectively (p=0.0001), at six months it was 55.77% ± 14.28% and 60.38% ± 19.79% respectively (p=0.0001). Following immediate post-training at 6 months, knowledge and skills scores increased to 94.91 ± 7.28% and 96.02 ± 4.50% respectively (p=0.0001). No participant had adequate knowledge and one had adequate skills at baseline. The proportion of those with adequate knowledge and skills markedly increased immediate post-training but decreased remarkably at three-month and at six-month evaluations respectively. 99.1% had adequate knowledge and all had adequate skills immediate post-training at 6 months.

**Conclusion:**

neonatal resuscitation training led to an improvement in knowledge and skills with suboptimal retention at three to six months post-training. Re-training improved knowledge and skills. We recommend that the retention of knowledge and skills could improve by retraining and mentoring at least 3-6 monthly.

## Introduction

The World Health Organization (WHO) estimated that globally, about 2.5 million newborns die during their first twenty-eight days of life each year [1,2]. The day of birth is the riskiest when nearly half of the newborn deaths occur. Majority of these newborn deaths are preventable and occur in developing countries [3,4]. Nigeria has the second-highest number of neonatal deaths in the world and the highest in Africa [2]. In 2017, the neonatal mortality rate in Nigeria was 36.6/1000 [5] and remains high, despite a decrease in other sub-Saharan African countries [6]. The major contributors to the high NMR include prematurity, birth asphyxia and sepsis [7-9]. Globally, birth asphyxia alone contributes 24% of all neonatal deaths, while in Nigeria, it accounts for 31% of neonatal deaths [10].

Neonatal resuscitation, which is a series of steps performed at the time of birth to help the newborn breathe, is a simple, low-cost intervention that has been shown to significantly reduce neonatal mortality from birth asphyxia by 20% - 30% [11,12]. The American Academy of Paediatrics (AAP) and its global partners developed neonatal resuscitation programmes such as Neonatal Resuscitation Programme (NRP) and Helping Babies Breathe (HBB) which offer standardized training to improve the knowledge and skills of newborn resuscitation among health care workers (HCWs) [13,14]. In sub-Saharan Africa, where over two-thirds of the world´s neonatal deaths occur [12], resuscitation is often not available for the majority of newborns, especially so, when deliveries happen at home or primary healthcare facilities. In these situations, birth attendants who are poorly skilled in neonatal resuscitation may perform practices that delay effective ventilation [15,16].

Nigeria, in an effort to reduce neonatal mortality rate incorporated neonatal resuscitation as one of the newborn interventions in the integrated maternal, newborn and child health strategy and ought to be practised at all levels of healthcare [17]. The Primary Health Centres (PHC centres) in Nigeria are run by Skilled Birth Attendants (SBAs) and offer health services including emergency obstetric and newborn care to a diverse group of pregnant women [18]. A proportion of these pregnant women had unsupervised antenatal care, delivered at home or in unorthodox centres [19,20]. Some of these cases are sometimes verbally sent to health centres after obstetric complications have arisen [21]. The competence of SBAs in neonatal resuscitation is hence crucial and has gained importance as a key ingredient to decreasing neonatal mortality [22]. Therefore, to ensure high coverage in resource-limited settings, training of SBAs in neonatal resuscitation merits priority [23,24].

Studies from West Africa, like Ghana and Sierra Leone, where neonatal resuscitation training was organized among HCWs, significant improvement in immediate post-training knowledge and skills were noted but retention after training was rarely documented [25,26]. Similarly, the implementation of Neonatal Resuscitation Training (NRT) program, the Nigerian adaptation of the AAP NRP, showed a gain in knowledge and skills among HCWs in secondary and tertiary centres in a few studies in Nigeria, although these studies did not assess retention [14,27]. Globally, there is growing concern whether HCWs retain the knowledge and skills learned after training in neonatal resuscitation, as loss of retention has been noted to occur 3 - 12 months after training but most of these studies were done in developed countries [26,28-31].

Despite the huge burden of neonatal deaths, there is limited information to suggest HCWs in PHC centres in Nigeria, West Africa have adequate knowledge and skills of basic neonatal resuscitation and retention has not been documented. The questions remain as to what happens to the retention and how frequently primary HCWs should be tested with regards to maintenance of competence in neonatal resuscitation after training.

## Methods

**Study area:** Rivers State is located in the southern part of Nigeria. It comprises of 23 local government areas (LGAs) and Port Harcourt Metropolis is the state capital which consists of Port Harcourt city LGA “PHALGA” and Obio-Akpor LGA. There are 28 PHC centres in Port Harcourt (13 in PHALGA and 15 in Obio-Akpor LGAs) that offer 24 hours obstetric and newborn services.

**Study design:** this was a prospective interventional study carried out before and after a one-day intervention.

**Study population:** the study population comprised of Health Care Workers (HCWs) including doctors and nurse/midwives who offer obstetric and newborn services at PHC centres in Port Harcourt.

**Sample size:** the sample size estimated to detect a minimum of 10% difference in mean knowledge and skills scores with 95% power at 95% confidence level using the formula for quantitative variables [32] was 106.

### Sampling

**Stage 1:** selection of PHC Centres: all 28 PHC Centres were included.

**Stage 2:** selection of HCWs: this was done by stratified sampling by proportionate allocation. One hundred and sixty eight HCWs were in both local government areas that provide obstetric and newborn services. In each PHC centre, the HCWs were stratified by cadre into doctors and nurse/midwives. Each facility had approximately two doctors and two to twelve nurse/midwives. Proportionate allocation was used to achieve the number of HCWs selected in each health facility. This was done using the formula: x/Ʃ_x_x n, where x=number of HCW in the health centre, Ʃx=total number of HCW in all the selected PHC centres and n=sample size of the study. For example, if a PHC centre had 5 nurses in the delivery room, the number that was randomly selected would be (5/168) x 106 = 3 nurses. Therefore, from the 28 PHC centres, the HCWs were selected using simple random sampling by balloting and a total of 106 HCWs were recruited. To minimise bias at each facility, the names of HCWs were selected from the monthly duty roster and numbers assigned to each name. Next, balloting was done by picking at random the required number to comprise the sample group as calculated by the proportionate allocation formula.

**Field researchers and assistants:** the researchers are certified NRP train-the-trainers facilitators of the paediatric association of Nigeria and have been involved in training at both state and national levels. The researchers facilitated all the modules and conducted both pre- and post-tests for knowledge and skills. Nine (9) volunteer field assistants certified in neonatal resuscitation were retrained. Each assistant had to obtain a passing score above 90%, in both knowledge and skills to be recruited.

**The NRT intervention:** the one-day training held on 18^th^ and 20^th^ of April, 2018 for two batches of 53 HCWs with a trainer: trainee ratio of 1: 5. Participants from the same facility were assigned to different batches to minimise bias and their absenteeism from work affecting patient care. Centres with more than two participants were batched accordingly. The training took place at the local government council hall. During the training, participants had didactic lectures and hands-on skills simulation using bag and mask for ventilating the neonatalie. The four modules taught included: overview of cardiopulmonary changes at birth, Initial steps in neonatal resuscitation, positive pressure ventilation and chest compression. These were adapted from the AAP NRP Textbook (6^th^ edition) [33].

### Study tools

**Tool for obtaining socio-demographic characteristics of health care workers:** a self-administered questionnaire was used to obtain information on the socio-demographics of participants, availability of neonatal resuscitation equipment at practising health centre, the average number of births per month and use of bag and mask in preceding six months.

**Knowledge assessment:** the HCWs´ knowledge was assessed pre and post-test using the best option, 18 multiple choice questions (MCQ), for modules 2-4 adapted from the NRP textbook [33] previously validated in Nigeria [14]. Knowledge assessment was considered adequate if the participant scored at least 80%.

**Skills assessment:** the HCWs´ skills were assessed by the Objective Structured Clinical Examination (OSCE) format, adapted from the NRP textbook [33] previously validated in Nigeria [14]. Skills were considered adequate with a minimum pass mark of 80% in the hands-on tasks, in addition to compulsory skill acquisition of bag and mask use and completion of all five critical steps (checks bag and mask and oxygen supply; indicates the need for positive-pressure ventilation; provides positive-pressure ventilation correctly; takes corrective action when heart rate not rising and chest not moving; demonstrates correct compression technique). The OSCE simulated the delivery of a term newborn in secondary apnoea, who required positive pressure ventilation, chest compressions with coordinated positive pressure ventilation after the ventilation correction steps had been performed to survive. Two points were awarded for every correct decision and properly performed skill. One point was awarded if the intervention was delayed or the technique for a given skill was inadequate. No point was awarded for indicated skills that were omitted or for performed skills that were not indicated. The sum of the awarded points was divided by the total possible points for that level of resuscitation multiplied by 100 to obtain a percentage score.

**Assessment of retention of knowledge and skills:** retention of knowledge and skills were assessed at three months (on the 24^th^ to 28^th^ July 2018) for 30 randomly selected HCWs and at six months (on the 22^nd^ and 23^rd^ October 2018) respectively, for all participants. The HCWs were not given prior notice before the evaluation at three months. All participants were contacted by bulk text messages before the six months assessment. Completion of the study was taken as HCWs who were followed up from baseline to six months post-training. Those who had unsatisfactory scores after initial training were re-trained and debriefed but initial post-test scores were documented. At the end of the initial training, sets of neonate specific bag and masks, reservoir bags and oxygen tubing were provided to the participating PHC centres where these were lacking, to enable participants to practice afterwards.

**Refresher training and assessment:** refresher training was done after the 6 months retention evaluation. All participants were again reassessed this was taken as “immediate post-training at 6 months” evaluation.

**Study procedure and data collection:** at presentation, each participant received a unique identification number and self-administered questionnaires were filled. Thereafter, baseline knowledge and skills were assessed. Researchers ensured proper spacing of participants together to avoid copying. The four modules were taught subsequently. Each module lasted 30 minutes. Demonstration of practice steps was following the NRP guidelines. Thereafter, participants practised each module for 30 minutes and were facilitated by researchers and assistants. They were also given an hour to revise the hands-on skills stations. Immediate post-test and skills evaluation were conducted afterwards. The entire session lasted for eight hours daily. Trainers´ ensured adequate time to all participants until learning was guaranteed, clarifying all questions per module.

**Statistical analysis:** data were analyzed using the Statistical Package for Social Sciences (IBM SPSS statistics), version 22.0 with the level of significance fixed at a p-value of <0.05. The demographic characteristics of the HCWs are displayed on tables. Continuous variables were expressed as means and standard deviations and categorical variables expressed as frequency tables, proportions and charts. Paired t-test was used to compare mean scores of knowledge and skills at two different evaluation times.

**Ethical approval:** the study was approved by the University of Port Harcourt Teaching Hospital ethics committee and permission from the permanent secretary of the Rivers State Primary Health Care Management Board was granted. Consent from each participant was obtained.

## Results

**Sociodemographic characteristics of health care workers that participated in the assessment:** one hundred and six HCWs participated in and completed the study. Nurses were 84 (79.2%) and the remaining twenty-two doctors were medical officers. Table 1 shows the sociodemographic characteristics of the HCWs. One hundred and three (97.2%) were females, with a female to male ratio of 34: 1. The mean age was 38.67 ± 8.14 years. The mean years of practice were 11.72 ± 9.22 years and ranged between one and 35 years.

**Table 1:** socio-demographic characteristics of the study participants

Variables	Frequency	Percentage
**Age category**		
≤30 years	17	16.0
31 - 40 years	57	53.8
41 - 50 years	20	18.9
>50 years	12	11.3
**Profession**		
Doctors	22	20.8
Nurses	84	79.2

**Distribution of health care workers´ experience on neonatal resuscitation:** Table 2 shows that three-eighths of the HCWs had previous training in newborn resuscitation. Half of these had neonatal resuscitation training within the previous three years. Six months before the study, about three-fifths of HCWs did not use a bag and mask during resuscitation while one-fifth had done so, on only one to four babies.

**Table 2:** distribution of study participants' experience on neonatal resuscitation

Variables	Frequency	Percentage
**Ever received training on newborn resuscitation (n=106)**		
Yes	40	37.7
No	66	62.3
**How long ago was last training received (n=40)**		
>1 - 3 years	20	50.0
4 - 6 years	12	30.0
>6 years	8	20.0
**Use of bag and mask (resuscitator) during newborn resuscitation**		
Yes	58	54.7
No	48	45.3
**Number of babies resuscitated using bag and mask in the past 6 months**		
None	65	61.3
1 - 4	22	20.8
5 - 8	13	12.3
9 - 12	1	0.9
>12	5	4.7

**Figure 1:**
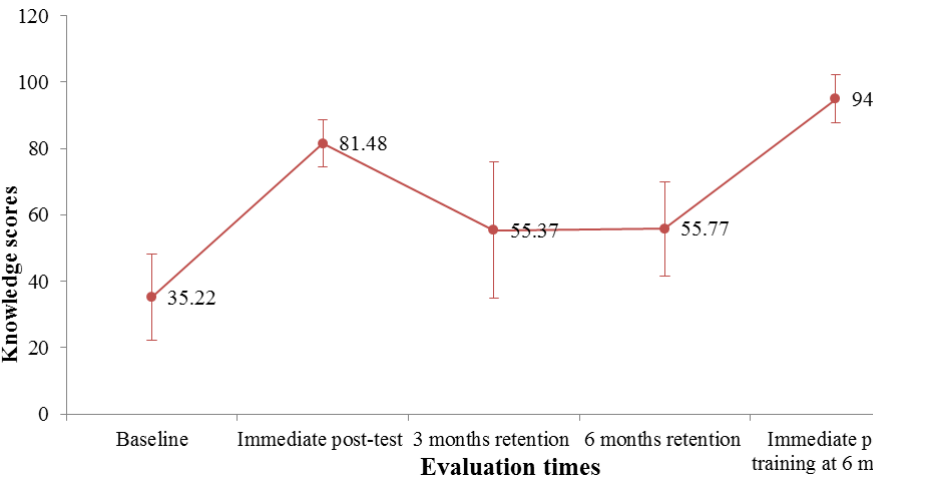
error bar chart of mean scores on knowledge of basic neonatal resuscitation at different evaluation times

**Figure 2:**
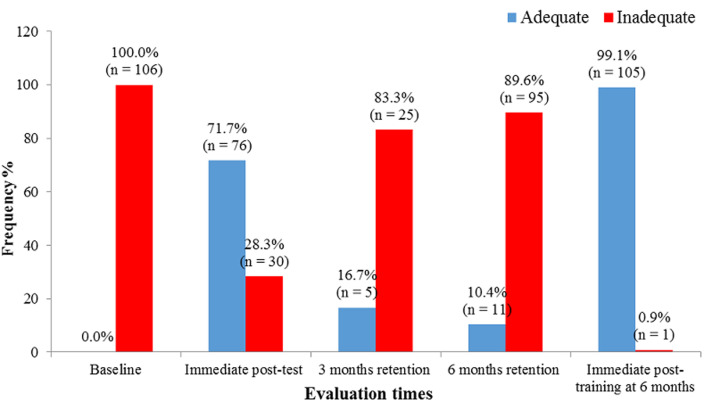
distribution of study participants' knowledge status of basic neonatal resuscitation at different evaluation times

**The trend of mean scores of knowledge and skills of basic neonatal resuscitation:** Figure 1 and Figure 2 show the trend of mean scores of knowledge and skills of neonatal resuscitation among HCWs. There was a statistically significant difference (p=0.0001) in mean scores of knowledge and skills across the different evaluation times as seen in Table 3 and Table 4 respectively.

**Table 3:** comparison of mean scores on knowledge of basic neonatal resuscitation at two different evaluation times using the paired t-test

Evaluation time/mean ± SD		Evaluation time/mean± SD		Paired t	p-value
Baseline (pretest)	35.22±12.90	Immediate post-test	81.48±7.05	-33.30	0.0001*
Immediate post-test	81.48±7.05	3 months retention	55.37±20.50	7.58	0.0001*
Immediate post-test	81.48±7.05	6 months retention	55.77±14.28	17.36	0.0001*
6 months retention	55.77±14.28	Immediate post-training at 6 months	94.91±7.28	-32.83	0.0001*

Statistically significant*; SD: standard deviation

**Table 4:** Comparison of Mean Scores on Skills of Basic Neonatal Resuscitation at two different evaluation times using the paired t-test

Evaluation time/mean± SD		Evaluation time/mean± SD		Paired t	p-value
Baseline (pretest)	21.40±16.91	Immediate post-test	87.40±13.97	-21.18	0.0001*
Immediate post-test	87.40±13.97	3 months retention	59.11±25.55	7.22	0.0001*
Immediate post-test	87.40±13.97	6 months retention	60.38±19.79	14.80	0.0001*
6 months retention	60.38±19.79	Immediate post-training at 6 months	96.02±4.50	-19.47	0.0001*

Statistically significant*; SD: standard deviation

**Figure 3:**
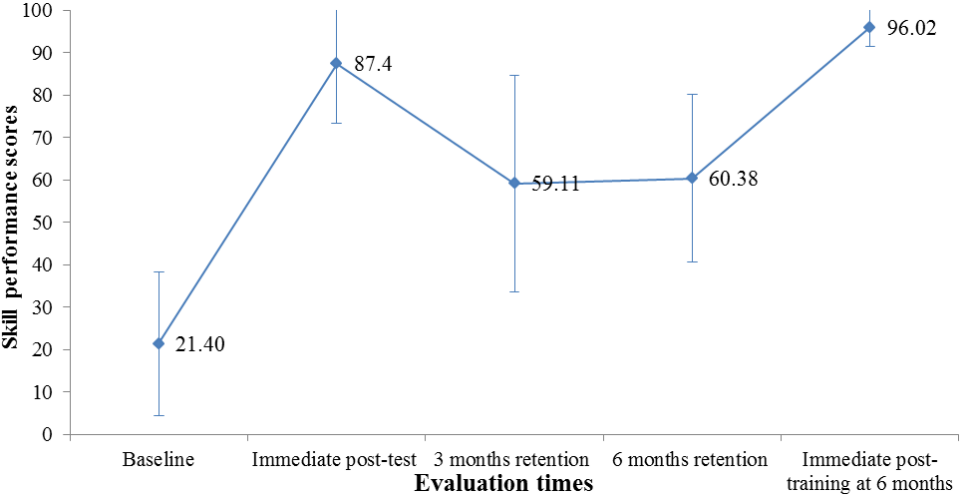
error bar chart of mean scores on skills of basic neonatal resuscitation at different evaluation times

**Figure 4:**
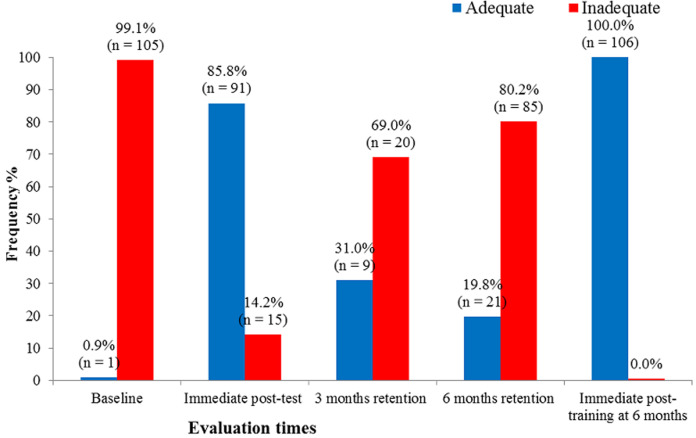
distribution of study participants' skills status on basic neonatal resuscitation at different evaluation times

**Knowledge and skills retention status:** Figure 3 and Figure 4 demonstrate that following a marked increase in the proportion of HCWs with adequate knowledge and skills (score above 80%) at immediate post-training, there was a steep and consistent decline in the proportion of HCWs with adequate knowledge and skills maintained at three and six months post-training respectively. After Immediate post-training at six months, almost all had adequate knowledge and skills status.

**Comparison of mean scores of knowledge and skills of basic neonatal resuscitation among doctors and nurses at different evaluation times:** the mean scores of knowledge of doctors were higher when compared to Nurses across the different evaluation times. This was only significantly higher at immediate post-test evaluation where the mean scores of knowledge of doctors were 82.32% ± 9.49% and nurses was 77.51% ± 8.83% (p=0.027). The mean scores of knowledge of nurses did not attain minimum pass cut-off when compared to doctors at immediate post-training evaluation. The mean scores of skills of doctors were consistently higher when compared to nurses across the different evaluation times. The difference in mean scores of skills was not statistically significant.

## Discussion

This study showed that the overall baseline knowledge and skills of neonatal resuscitation among HCWs in PHC centres in Port Harcourt were inadequate. Similar observations have been made in other studies in Nigeria and other developing countries [14,25,27,34-38]. The general dearth of knowledge and skills level among the HCWs in this study was most striking. The finding that all the HCWs had inadequate knowledge and 99.1% had inadequate skills at baseline was similar to findings in other Nigerian studies by Oloyede *et al*. [27] and Umar *et al*. [38]. Some neonatal resuscitation training was known to have occurred among HCWs in a tertiary facility in Port Harcourt [39]. However, there is no known formal basic neonatal resuscitation training among primary HCWs in Port Harcourt. The findings in this current study were in contrast to findings in Pakistan [37] where four-fifths of the 'lady health workers' and half of the midwives answered correctly to questions that assessed knowledge of newborn resuscitation.

The much lower baseline knowledge and skills scores of HCWs in this study could be due to the lack of simulation-based training or refresher training as the few who had previous training did so in the previous 3-6 years. It could also be due to inadequate/unavailability of resuscitation equipment such as bag/masks and lack of exposure to an adequate number of real cases requiring neonatal resuscitation as most respondents reported to have had very few cases that required bag-mask ventilation in the six months before this study. Furthermore, the lack of certification process after pre-service training among these HCWs may also be a contributing factor. In contrast, in the study in Pakistan, the display of higher knowledge by the primary HCWs could be because they had extensive pre-service and refresher training with an emphasis on both the theory and practical aspects of basic neonatal resuscitation. There was also better availability of neonatal resuscitation equipment in the PHC centres in the Pakistan study which could have aided the performance of effective resuscitative actions. The similar pretest scores amongst the doctors and nurses in this study may be reflective of the general lack of knowledge and training of basic neonatal resuscitation among HCWs in primary health facilities. The finding in this study was comparable to the results obtained in the studies by Disu *et al*. [14] Oloyede *et al*. [27] and Umar *et al*. [38] in Nigeria but differ from a study in Ghana, by Enweronu-Laryea *et al*. [25] where nurses and midwives demonstrated lower pretest knowledge scores compared to doctors. This may reflect a lack of emphasis on neonatal resuscitation during pre-service training in Nigeria.

Immediately after NRT intervention, there was a significant improvement in knowledge and skills among the primary HCWs. The higher immediate post-test compared to pre-test scores for all HCWs confirms that there was an acquisition of knowledge and skills after the intervention. The finding is consistent with other studies in developing countries demonstrating NRP increased acquisition of knowledge and skills [14,25,27,31,35,36,38,40].

In this study, the immediate post-test knowledge scores were significantly higher among doctors than nurses but not for the post-test skills scores. This difference, however, was no longer observed at six months post-training assessment. Studies in Nigeria by Umar *et al*. [38] and Oloyede *et al*. [27] showed similar results; however, no significant difference was documented between doctors and nurses in a Canadian study by Skidmore [41]. The finding in this present study may reflect the inherent differences in the pre-service curriculum of doctors, bearing in mind the difference in scope and depth of undergraduate curriculum which makes an understanding of pathophysiologic concepts less tedious for doctors compared to nurses. However, in the study by Skidmore [41], the repetitive training sessions that occurred several times a week may have accounted for their finding.

The study demonstrated that at six-month retention assessment, the HCWs retained some learned knowledge and skills though with a significant decline in their scores. This decline had already been documented at three months post-training in a subset of the HCWs. Also, there was a consistent and steep drop in the proportion of the HCWs that had 'adequate' knowledge and skills at three and six months post-training respectively. This is important because even after achieving the minimum pass cut-off, most of the HCWs without 'an intervention' within three to six months became 'unqualified' to perform neonatal resuscitation. This implies that despite initial training, about 90% of the HCWs did not retain the knowledge and skills and were more likely to carry out substandard resuscitative practices. In other words, they were no longer 'qualified' to resuscitate newborns by all standards of practice. The findings in the current study are similar to those obtained in several studies which demonstrate that knowledge and skills drop as early as three to six months after training [26,42]. However, this contrasts the study by Das *et al*. [36] among Indian doctors and nurses who had maintained similar scores for both knowledge and skills at 12 months post-training. The apparent longer retentive capabilities occurred because the HCWs in the study by Das *et al*. [36] were exposed to supportive supervision and skills laboratories that provided the opportunity for more hands-on practice and peer-learning. The findings in this present study, therefore, suggest that a one-off training may not be suitable among these cohorts of HCWs.

The suboptimal knowledge and skills scores demonstrated at six-month retention evaluation were however, improved upon at an immediate post-training at six months evaluation, with participants obtaining significantly higher scores than was earlier documented at immediate post-test six-months earlier. In the immediate post-test, the midwives/nurses did not attain the minimum cut-off. This finding is consistent with a study in Ghana [25]. It is plausible that midwives/nurses in PHC centres may benefit from a modified basic neonatal resuscitation training such as the HBB curriculum. However, the findings in this study showed that after refresher training the midwives/nurses attained the minimum cut-off. This highlights the fact that irrespective of the curricular used for training, refresher training is necessary to sustain knowledge and skills in basic neonatal resuscitation. The advocacy for refresher training with mentoring to support knowledge and skills retention has been proposed by some studies [36,40]. Our findings, therefore, have identified a gap in knowledge and skills retention among primary HCWs and suggest that to ensure optimal knowledge and skills in basic neonatal resuscitation, regular retraining could be key to maintain optimum levels of competence.

**Limitation:** the researchers were not blind while evaluating skills pre/post-training. However, because the evaluation was with a checklist for skills and had specific scoring instructions, the potential for bias was limited. Appointments for post-testing at 6 months evaluation was necessary; therefore, participants may have reviewed lecture notes beforehand resulting in better scores. This could not be controlled for in this study. However, at 3 months, participants were not given prior notification before evaluation. Assessment of HCWs response to real-life situations was not possible since mannequin simulation was used in this study.

## Conclusion

The effect of neonatal resuscitation training on neonatal outcomes eventually depends on how much knowledge and skills are learned, retained and correctly applied to newborns when the need arises. Our study highlights a high loss of retention among primary health care workers at three to six months and suggests the need for at least a three to six monthly refresher training among this cohort of health care workers.

### What is known about this topic

- Birth asphyxia remains a major contributor to neonatal mortality in resource-limited settings like Nigeria;
- Birth attendants skilled in newborn resuscitation are necessary to prevent asphyxia related complications;
- Studies which assess knowledge and skills of basic neonatal resuscitation among primary health care workers in Nigeria are limited.

### What this study adds

- Confirms a limited knowledge and skill of basic neonatal resuscitation among primary health workers;
- Highlights that one-off training of primary health workers in neonatal resuscitation is not sufficient;
- Training and retraining are vital to maintaining optimal knowledge and skills in neonatal resuscitation.
